# A protocol for developing, disseminating, and implementing a core outcome set for caesarean scar ectopic pregnancy research: COSCAR

**DOI:** 10.1186/s13063-025-08879-7

**Published:** 2025-06-04

**Authors:** Simrit Nijjar, Cecilia Bottomley, Munira Oza, Ilan E. Timor-Tritsch, Andrea Kaelin Agten, Francesco D’Antonio, Krystle Y. Chong, Lan N. Vuong, Jin Li, Rosanna Acklom, Ben W. Mol, Davor Jurkovic, the International Collaboration COSCAR

**Affiliations:** 1 EGA Institute for Women’s Health, Faculty of Population Health Sciences, University College London, London, UK; 2 The Ectopic Pregnancy Trust, London, UK; 3 Hackensack Meridian School of Medicine, Nutley, NJ USA; 4 Liverpool Women’s Hospital NHS Foundation Trust, Liverpool, UK; 5 Center for Fetal Care and High-Risk Pregnancy, Department of Obstetrics and Gynecology, University Hospital of Chieti, Chieti, Italy; 6 Department of Obstetrics and Gynaecology, Monash University, Clayton, Australia; 7 Department of Obstetrics and Gynecology, University of Medicine and Pharmacy at Ho Chi Minh City, Ho Chi Minh City, Vietnam; 8 Department of Obstetrics and Gynecology, National Clinical Research Center for Obstetric and Gynecologic Diseases, Peking Union Medical College Hospital, Chinese Academy of Medical Sciences and Peking Union Medical College, Beijing, 100730 China; 9 Monash Women’s, Monash Health, Clayton, Australia

**Keywords:** Caesarean scar ectopic pregnancy, Treatment, Modified Delphi methodology, Core outcomes

## Abstract

**Background:**

Caesarean scar ectopic pregnancy (CSEP) is the most common of type of uterine ectopic pregnancy and is associated with significant morbidity. Prompt diagnosis and treatment is therefore of paramount importance. Currently there is no universally agreed treatment option for CSEP supported by any national or international society. Studies evaluating CSEP management report many different outcomes and often define and measure success or complications of various treatments in different ways. This variation in reporting of outcomes leads to heterogeneity and an inability to directly, or reliably compare results of studies, leaving the question of what the optimal treatment is unanswered. We aim to develop a minimum set of outcomes that should be reported in all future research in CSEP.

**Methods:**

An international steering committee of key stakeholders, including researchers, healthcare professionals, patient advocates, and people with a lived experience of CSEP, has been established. A long list of potential outcomes will be identified from a systematic literature review and by interviewing people with a lived experience of CSEP. Key stakeholders will then be asked to prioritise the outcomes via a modified 2-round Delphi survey. Outcomes will be scored using a modified nine-point Likert scale that ranges from 1 (extremely unimportant) to 9 (extremely important) and an additional outcome of ‘I can’t rate the outcome because I don’t know the outcome’. Finally, the steering group will refine by consensus the final core outcome set. The consensus process will result in a core outcome set that is internationally relevant to all key stakeholders. We will actively disseminate our findings to help improve clinical trials and guidelines with the ultimate aim of improving the diagnosis and management of CSEP.

**Discussion:**

Implementing a core outcome set for CSEP will prevent research waste and improve patient centredness, by enabling reliable comparisons of different treatments for CSEP. This process will also help raise awareness of this condition, increasing clinician knowledge, which in turn will help them counsel patients more effectively, therefore benefiting professionals and patients alike. Expertise in diagnosing and managing this condition is currently focused in a handful of expert centres and many healthcare professionals are not always confident or comfortable in managing these patients and therefore refer them to other centres, which can be considerable distances from patients’ localities. This core outcome set will aim to advance sharing of knowledge and spread expertise in time.

**Trial registration:**

COMET 2903. Registered in November 2023. Available online on: https://www.comet-initiative.org/Studies/Details/2903.

**Supplementary Information:**

The online version contains supplementary material available at 10.1186/s13063-025-08879-7.

## Background

Caesarean scar ectopic pregnancy (CSEP) is the most common type of uterine ectopic pregnancy and is defined by implantation of the pregnancy into a myometrial defect caused by dehiscence of a previous lower uterine segment caesarean scar [1]. CSEPs that continue after diagnosis with the aim of a live birth are associated with increased foetal loss and morbidity from severe prematurity and also significant maternal morbidity, particularly massive obstetric haemorrhage (MOH), need for blood transfusion, placenta accreta syndrome (PAS), uterine rupture, emergency hysterectomy, and maternal death [2–4]. However, even failed CSEPs are at risk of major bleeding, need for blood transfusion, and emergency hysterectomy [2]. The general consensus is that CSEPs should therefore be managed in the early first trimester to reduce morbidity [3, 4]. The potential for CSEP to result in live birth in a significant number of cases has triggered debate regarding whether it can be classified as a true ectopic pregnancy [5, 6]. However, CSEP is associated with the risk of severe bleeding due to the pregnancy being implanted partially or completely outside the uterine cavity. In view of this, several organisations, including the Royal College of Obstetricians and Gynaecologists, the European Society of Human Reproduction and Embryology, and the Society for Maternal–Fetal Medicine (SMFM), all classify caesarean scar pregnancy as an ectopic pregnancy in their guidelines [7–9].

Despite the impact of this condition, there is no universally recommended treatment option for CSEP supported by any national or international society, with the SMFM concluding that the optimal treatment option remains unknown but strongly recommending (based on moderate quality evidence) against expectant management of CSEPs [10]. This uncertainty of treatment likely relates to the heterogeneity in reported outcomes and publication bias, with a significant proportion of studies published from a relatively small number of countries [2]. Treatment falls into four main categories, expectant management (of a failing pregnancy or of a continuing pregnancy), medical management (local or systemic methotrexate), surgical management (suction curettage versus resection by multiple routes), and other management (uterine artery embolisation, high-intensity focused ultrasound, local sclerotherapy, and Foley balloon catheter). Multiple treatment regimens and combinations have been reported in the literature, with one review including 751 patients and identifying over 30 different treatments [11]. These studies are predominantly case series of varying number of cases and quality, with a limited number of randomised controlled trials (RCTs) [12–16]. Case studies are generally considered very low-level evidence and therefore provide a lower confidence that the evidence reported reflects a true effect. These studies report many different outcomes and often define and measure success or complications of various treatment modalities in different ways [2]. For example, the insertion of a Shirodkar suture following suction curettage was considered a complication in one review, but as part of the planned management in another study [6, 12]. Studies also define success of a treatment diversely, from no retained pregnancy tissue to no additional treatment required. This variation in reporting of outcomes leads to heterogeneity and restricts effective data synthesis, leading to an inability to compare results of studies directly or reliably, ultimately resulting in bias in outcome reporting, which impacts the ability to inform clinical practice [2].

A core outcome set (COS) for research in ‘Ectopic Pregnancy’ was published in 2023, which was primarily focused on tubal ectopic pregnancies [17]. However, CSEP is a distinct condition in comparison to all other ectopic pregnancies, both in terms of pathophysiology, the natural progression of the condition, and associated morbidity; thus, although some of the outcomes from the published ‘Ectopic Pregnancy’ COS are relevant to the CSEP population, a distinct COS needs to be developed for CSEP to include important outcomes that were not addressed in that publication. For example, CSEP is the only type of ectopic pregnancy where a significant proportion of pregnancies may progress to viability (albeit often with significant morbidity) and therefore treatment options can include termination of a potentially viable pregnancy (which is not usually offered to any other type of ectopic pregnancy) or expectant management with the potential of a live healthy baby. CSEP is associated with both maternal morbidity (loss of future fertility, uterine rupture, bladder injury, obstetric hysterectomy, PAS) and foetal morbidity (second trimester loss, prematurity, neonatal morbidity). As expectant management is one treatment option for CSEP, any COS for CSEP must include the aforementioned outcomes that occur beyond the first trimester. In comparison, other ectopic pregnancies do not generally progress beyond the first trimester, so these late outcomes are not covered by the previously published COS for ‘Ectopic Pregnancy’ [17]. We therefore propose developing an add-on COS for CSEP, which will take the overarching COS for ‘Ectopic Pregnancy’ as default core outcomes (where they are applicable to CSEP) and also seek any additional core outcomes for research in CSEP. Evidence shows that COS developed for other conditions are now effectively being translated into research practice [18]; this change will ultimately lead to improved clinical outcomes for patients.

The aim of this study is to develop a COS to ensure outcomes important to all key stakeholders are measured and reported consistently in all future research studies investigating all treatments of CSEP, which will allow for a more reliable head-to-head comparison of the different treatment modalities and their outcomes. This will then enable synthesis of high-quality data to inform evidence-based treatment guidelines for CSEP.

## Methods

**Fig. 1:**
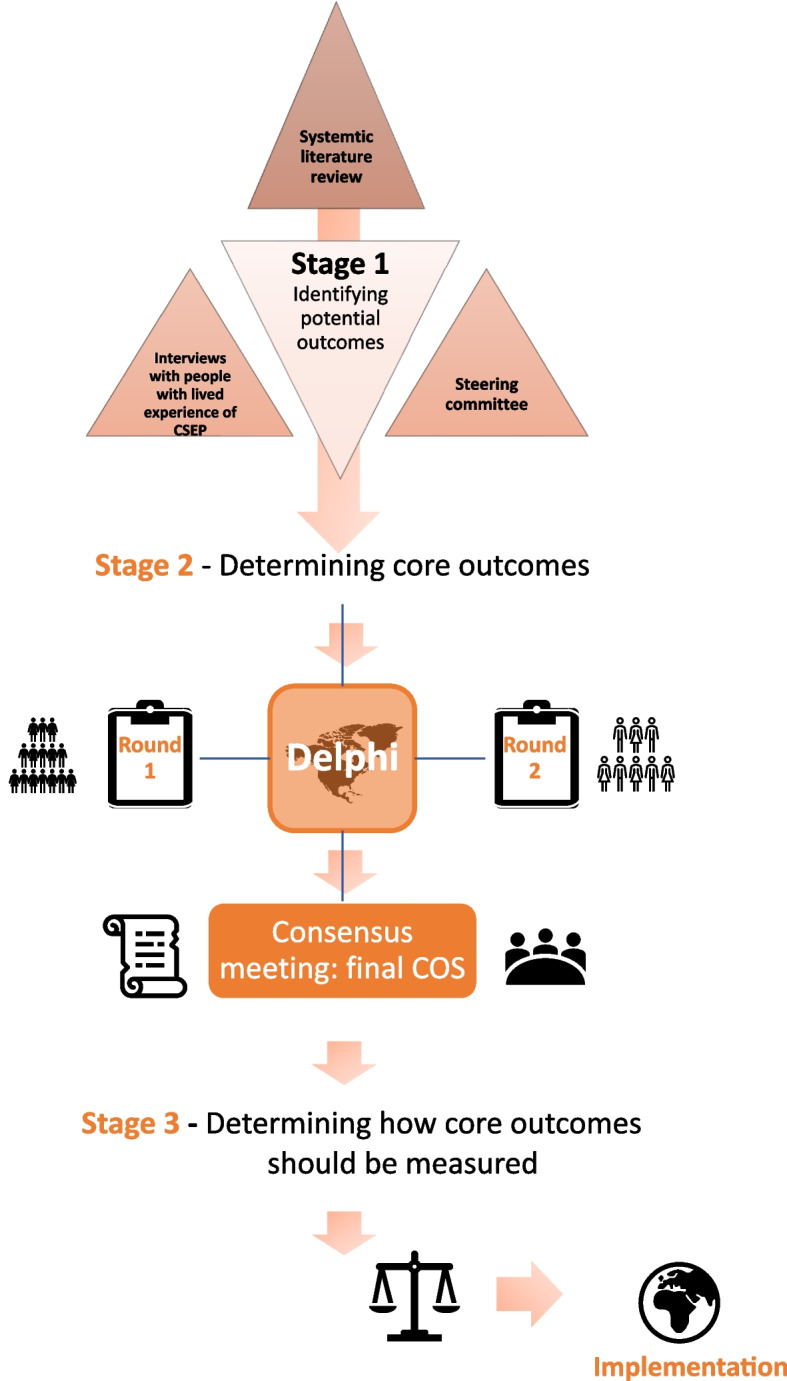
Overview of the stages in the development of the COS for CSEP research. (This protocol does not cover stage 3)

The methods for COSCAR have been informed by the Core Outcome Measured in Effectiveness Trials (COMET) initiative handbook [19]. A mixed-method consensus approach will be used to create the COS, using a modified Delphi process and consensus meetings. This is in line with protocols successfully implemented in previous COS studies [17, 20]. Figure 1 illustrates the stepwise methodology that will be required to develop a COS for CSEP research. To ensure the success and credibility of the COS, we will ensure engagement of key stakeholders (researchers, healthcare professionals, patients, and patient representative organisations) and adhere to the recommendations of the COS-STAP and COS-STAR statements [18, 21].

**Fig. 2:**
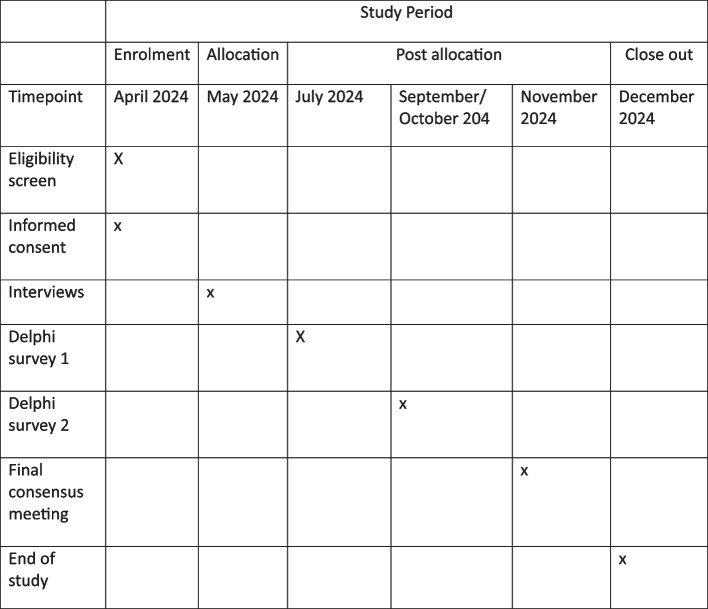
Schedule of enrolment and interventions

The primary objective of this project will be to develop a minimum set of outcomes to be reported in all future studies that investigate any treatment intervention for CSEP. Secondary objectives include engaging patients and the public in CSEP research, to achieve an increased awareness of this not so well-known type of uterine ectopic pregnancy (Fig. 2).

### Statistical analysis

Proportions of agreement for outcomes will be calculated for all participants and each stakeholder group separately using SPSS version 28.0.1.1 (IBM Corporation, Armonk, USA).

### Study organisation

#### Managing team

This team will have oversight of the study including co-ordinating meetings of the steering group, conducting the literature review, interviewing people with a history of CSEP, developing the long list of outcomes, conducting the Delphi survey, and organising the consensus meeting. They will prepare all publications resulting from the study.

#### Steering group

This will consist of the managing team and several international experts in CSEP. The steering group will include healthcare professionals, researchers, individuals with a lived experience of CSEP, and representatives from patient advocacy groups. We will aim for the steering group to be made up of no more than 15 individuals.

#### Steering advisory group

The advisory committee will consist of experts and patient representatives recommended by the steering group. The steering advisory group will guide the narrative of the Delphi by reviewing the proposed categorisation and description of outcomes, reviewing the long list of outcomes prior to the Delphi survey, and approving the final list following the consensus meeting.

#### Key stakeholders

Stakeholders falling into three key groups will be invited to participate in this study. Input from all these groups is required for this COS to be credible and for the outcomes to be truly reflective.

- Researchers in CSEP.
- Healthcare professionals that diagnose and treat women with CSEP.
- Women or partners with a lived experience of CSEP and patient representative organisations.

### Scope of this core outcome set

This COS will apply to all studies, systematic reviews, and clinical guidelines evaluating interventions for women with CSEP. We will not limit the COS to a certain study type given the relative rarity of CSEPs, 1.5 per 10,000 maternities [22]. The COS will be defined for all treatment options for CSEP, including expectant, medical, surgical, and other management modalities. Recommendations for the diagnosis of CSEP in studies are not within the scope of this COS and will require a separate process.

### Identifying potential core outcomes

A long list of outcomes will be identified through a comprehensive systematic literature review of studies reporting management of CSEP. We will extract outcomes using the wording in the published papers verbatim. We will then group the similar outcomes into outcome domains, which will broadly classify different aspects of treatment effect [19].

Additional potential outcomes will be identified by conducting semi-structured clinical interviews with individuals with a lived experience of CSEP. New outcomes identified from this process will be added to the long list of outcomes generated by the systematic review. Given the relatively low prevalence of CSEP and to ensure we have sufficient engagement from patients for development of the long list, we will also liaise with patient advocacy groups to conduct a survey of their members to see what further potential outcomes are important to them.

As this COS for CSEP is an add-on COS for the already published COS in ‘Ectopic Pregnancy’ [17], the managing team will determine which default core outcomes should be included from the existing COS, based on their applicability to CSEP (Table 1).

**Table 1:** Core outcomes presented from the pre-existing ‘Ectopic Pregnancy’ COS [17]

Core outcome in Ectopic Pregnancy COS	Definition
Treatment success (including reasons for initial treatment failure)	Defined according to location of EP a. Extrauterine EP (tubal/ovarian/abdominal): resolution of β-hCG to prepregnancy levels, or after definitive surgical removal of EP, using the initial treatment protocol **b. Uterine EP (cervical/intramural/caesarean scar/rudimentary horn): resolution of trophoblast tissue on ultrasound, using the initial treatment protocol** **Reason for initial treatment failure** **a. Clinical urgency: including pain, infection, and haemorrhage** **b. Clinician choice: use of additional intervention because of prolonged resolution of EP, as detected by persistent β-hCG or trophoblast** **c. Patient choice**
Resolution time of EP	a. Extrauterine EP: number of days until β-hCG returns to prepregnancy levels **b. Uterine EP: number of days until trophoblast seen on ultrasound is no longer visualised**
No. of additional interventions	**a. As per protocol (forming part of routine treatment)** **b. Out of protocol (forming part of rescue treatment) including additional medical treatment after surgery**
Adverse events	a. **Medication complications: complications related to medical therapy including adverse side effects of methotrexate** b. Surgical complications specific to EP i. Removal of an incorrect fallopian tube ii. Removal of an ovary **iii. Hysterectomy** **iv. Persistent EP (persistent β-hCG or trophoblast)** v. Failed salpingotomy resulting in salpingectomy **c. General surgical complications** **i. Conversion from laparoscopy to laparotomy** **ii. Return to theatre** **iii. Venous thromboembolism** **iv. Sepsis** **v. Blood loss** **vi. Anaesthetic complications** d. Complications from EP (including expectant management) i. Tubal rupture **ii. Psychological distress**
Mortality and severe morbidity	**Confirmation of death, admission to intensive care unit, or major blood loss requiring blood transfusion**
Treatment satisfaction	**Patient satisfaction with care received including treatment and care providers**

Outcomes highlighted in bold are the ones we propose to include in our new COS for CSEPs

*β-hCG *beta–human chorionic gonadotropin, *EP *ectopic pregnancy

### Identifying stakeholders

Several strategies will be employed to identify and optimise contributions from key stakeholders, including use of existing national and international clinical and research networks and advertising at relevant scientific meetings. We will also directly contact editors of scientific journals in gynaecology to ask them to distribute the survey invitation to their readers. We will identify corresponding authors of CSEP studies through literature review and contact them directly.

Other strategies that will be employed will be development of a website containing information on the study and a link to register interest in participation (www.coscar.org). We will also liaise with patient advocacy groups and charities and ask them to distribute the invitation for the study to their followers through their social media platforms and mailing lists. We will also directly approach individuals involved in the clinical care or research of CSEP and ask them to advertise the survey link to patients who have experienced CSEP, in their clinics or on their social media platforms. All stakeholders will receive email invitations with a link to access the online Delphi survey.

#### Group size

There is no statistical methodology to determine the optimum number of stakeholders to include in the Delphi process and rather group sizes should be determined by the rarity of the condition [19]. With this in mind, we will be aiming for at least 10 participants in each stakeholder group but will aim to recruit as many individuals from each stakeholder group as possible. To mitigate for the potentially smaller stakeholder group sizes, we will try and ensure there is good representation of qualified experts with a deeper understanding of the issues in managing CSEPs in the clinical and research stakeholder groups [19].

### Selecting core outcomes by consensus

#### Delphi process

The long list of outcomes will be inputted into the Delphi survey, which will be piloted on several members of the steering group and a sample of stakeholders, including individuals with a lived experience of CSEP, to ensure its feasibility and accessibility. Survey questions containing medical terminology will be accompanied by plain English explanations to enhance understanding. We will collaborate with a patient advocacy organisation to ensure clarity and provide study materials tailored for all stakeholder groups on our study website, www.coscar.org, including patient-friendly information. All stakeholders that respond to the invitation to participate (or register on the website for the study) will be emailed an electronic link to the Delphi survey. The final long list of outcomes will be presented in a modified 2-round Delphi process. Participants will rank priorities in the first round and be invited to suggest additional outcomes for the second round. For the second round, participants will have the opportunity to modify initial rankings after having sight of cumulative responses from fellow stakeholders. For each survey completed, we intend to offer a contribution to a registered early pregnancy charity.

At the start of the survey in both rounds, participants will be asked to provide demographic details and will be provided signposts to The Ectopic Pregnancy Trust and other support routes. They will also be provided with a unique identifier to facilitate responses in future rounds with anonymity. We anticipate and will encourage local translation of the Delphi surveys to allow non-English speakers to be able to contribute.

#### Round 1

In round 1, the long list of outcomes will be presented in a Delphi survey using the online survey platform, REDCap, which we hope will optimise global representation of all stakeholder groups. Outcomes from the long list will be presented in individual domains and stakeholders will be asked to score individual outcomes using a modified nine-point Likert scale which has been widely accepted as a valid methodology for reviewing outcomes in COS [23, 24]. The scale usually ranges from 1 (not important for decision-making) to 9 (critical for decision-making). We will additionally include a category, ‘I can’t rate the outcome because I don’t know the outcome’, as some stakeholders may feel they do not have the expertise to score a certain outcome [19]. In addition to the Likert scale, participants will be provided with a written explanation of what the numbers correspond to on the scale to minimise confusion and error [20].

Participants will have a 4-week window in which to complete the survey. At the end of round 1, outcomes will be summarised for all, by stakeholder group and individually. Any additional outcomes will be reviewed by the steering group and included in round 2 if felt to be appropriate. Only participants who fully complete round 1 will be invited to round 2.

#### Round 2

In round 2, participants will be asked to review outcomes that did not reach consensus in round 1 and any additional new outcomes suggested by stakeholders in round 1. The outcomes that reached consensus in round 1 will be presented in round 2 but will not be up for vote again. This approach enables prioritisation of outcomes that have less agreement in round 1 [19]. Participants will be shown the number of respondents, distribution scores for each outcome summarised by each stakeholder group and their own score. To increase consensus, participants are expected to reflect on the feedback from other stakeholders before re-rating outcomes on the modified nine-point Likert scale, as they may wish to change their score in round 2 based on the group’s opinions [19].

Those outcomes that do not reach a consensus in round 2 will be then discussed at the stakeholder consensus meeting. The second round of the Delphi survey will be held open for 4 weeks; however, this may be extended if uptake is low.

#### Stakeholder consensus meeting

After the 2-round Delphi process, a consensus meeting with the steering group will be conducted to review the list of core outcomes thought to be potentially important with the objective of developing the final COS for CSEP. At the conclusion of the second round, stakeholders will have the opportunity to indicate their interest in participating in the consensus meeting by responding at the end of the survey. A small sample of stakeholders will then be invited to participate, to ensure fair representation from all stakeholder groups. At the consensus meeting, each outcome will be summarised again, both individually and by stakeholder group, and provided to the participants prior to and during the consensus meeting. During the meeting, each outcome will be reviewed and those that do not reach consensus through the Delphi survey will be discussed and voted on by the meeting participants. Voting will occur anonymously online. If this meeting does not result in consensus, a further Delphi round or a majority vote on the item will be proposed.

The steering group and other key stakeholders will be invited to attend the meeting. Individuals will be selected to be invited to the meeting using the following principles to ensure good representation: participants have completed both rounds of the survey (we will include a final question at the end of the round 2 survey about willingness to participate in a consensus meeting), a balance in numbers across all 3 stakeholder groups, and reasonable geographic spread. We anticipate the latter two criteria may be more difficult to achieve as there are likely to be fewer patients (given CSEP is not common) compared to clinicians, and researchers tend to be from a small number of countries. If an individual cannot attend, they will be replaced by someone from the same stakeholder group whenever possible.

We will aim for this meeting to be a hybrid meeting, conducted in person in London, UK and virtually, to maximise participation and optimise global representation from all stakeholder groups to reduce bias.

### Defining consensus

A priori consensus definitions will be used to assess agreement and disagreement between individual stakeholders and stakeholder groups. Consensus that an outcome should be included in the COS will be defined as 70% or more of participants scoring it as 7–9 and less than 15% of participants scoring it as 1 to 3 [18]. Consensus that an outcome should not be included in the COS will be defined as 70% or more of participants scoring it as 1 to 3 and less than 15% of participants scoring it as 7 to 9. In outcomes where the aforementioned criteria are not reached, it will be deemed that consensus is ‘equivocal’. The outcomes that are designated as ‘consensus in’ by all stakeholder groups in both rounds will be included in the final core outcomes to be carried forward to the consensus meeting. If an outcome is designated ‘consensus in’ by one stakeholder group but not the others, the item will be discussed in the consensus meeting.

#### Missing data

Participants will be required to rate every outcome. To minimise incomplete data from individual participants, survey respondents will have to rate each outcome before they can proceed to rate the next outcome. Survey respondents will have the option of saving, leaving, and then going back to complete the survey later. If the survey is not completed by a participant, we will send them at least two reminder emails to complete it, if they do not complete it, part completed surveys will not be included in the analysis. To minimise attrition bias, we will aim to obtain answers from at least 80% of participants in each stakeholder group [19]. To assess for attrition bias, we will analyse the answers provided by participants in round 1 only comparing them to those completed in both rounds [25].

#### Ethical approval

Ethical committee approval (UK NHS Health Research Authority Research Ethical committee approval reference 24/LO/0190) was obtained prior to the start of this study. Informed consent will be assumed if a participant completes the Delphi survey or attends the consensus meeting. All participants involved in the clinical interviews will be asked for written consent.

### Prospective registration

This study has been prospectively registered with the Core Outcome Measures in Effectiveness Trials (COMET) initiative, the registration number is 2903 and is available online on: https://www.comet-initiative.org/Studies/Details/2903.

## Discussion

### Dissemination and implementation

To maximise dissemination and uptake of the final COS, we will publish it in a peer-reviewed scientific journal and present the COS at scientific meetings. The Core Outcomes in Women’s Health (CROWN) initiative will also encourage uptake of the COS by researchers. This group is made up of journal editors in women’s health and their aim is to promote the development of core outcomes in obstetrics and gynaecology; strongly encourage researchers to report the results of established COSs and ensure effective dissemination of the COS [26].

Of the 108 studies identified in our literature review, over three hundred different outcomes were reported across six domains. Development of this COS will promote more standardised reporting and measuring of outcomes in CSEP treatment, therefore allowing healthcare professionals to deliver more evidence-based care to women.

## Conclusion

This COS in CSEP will not only inform future research and synthesis of meta-analyses but will also guide the development of much needed clinical guidelines. Adoption of this COS will ultimately lead to improved patient-centred care and outcomes.

## Trial status

This protocol is version number 1.1, dated on 20 th March 2024. Recruitment began in April 2024, and the three rounds of the consensus process is anticipated to be completed by January 2025.

## Supplementary Information

Additional file 1. SPIRIT checklist

## Data Availability

Available on request from corresponding author.

## Abbreviations

COMET: Core Outcome Measured in Effectiveness Trials
COS: Core outcome set
COSCAR: Core outcome set for caesarean scar ectopic pregnancy research
COS-STAP: Core Outcome Set-STAndardised Protocol Items
COS-STAR: Core Outcome Set–STAndards for Reporting
CROWN: Core Outcomes in Women’s and Newborn Health
CSEP: Caesarean scar ectopic pregnancy
MOH: Massive obstetric haemorrhage
PAS: Placenta accreta syndrome
RCTs: Randomised controlled trials
SMFM: Society for Maternal–Fetal Medicine
